# Evolution of Epiphytism and Fruit Traits Act Unevenly on the Diversification of the Species-Rich Genus *Peperomia* (Piperaceae)

**DOI:** 10.3389/fpls.2016.01145

**Published:** 2016-08-09

**Authors:** Lena Frenzke, Paul Goetghebeur, Christoph Neinhuis, Marie-Stéphanie Samain, Stefan Wanke

**Affiliations:** ^1^Department of Biology, Institut für Botanik, Technische Universität DresdenDresden, Germany; ^2^Department of Biology, Research Group Spermatophytes, Ghent UniversityGent, Belgium

**Keywords:** BaMM, BiSSE, diversification, dispersal, epiphytism, epizoochory, fruit morphology, life form

## Abstract

The species-rich genus *Peperomia* (Black Pepper relatives) is the only genus among early diverging angiosperms where epiphytism evolved. The majority of fruits of *Peperomia* release sticky secretions or exhibit hook-shaped appendages indicative of epizoochorous dispersal, which is in contrast to other flowering plants, where epiphytes are generally characterized by fruit morphological adaptations for anemochory or endozoochory. We investigate fruit characters using Cryo-SEM. Comparative phylogenetic analyses are applied for the first time to include life form and fruit character information to study diversification in *Peperomia*. Likelihood ratio tests uncover correlated character evolution. We demonstrate that diversification within *Peperomia* is not homogenous across its phylogeny, and that net diversification rates increase by twofold within the most species-rich subgenus. In contrast to former land plant studies that provide general evidence for increased diversification in epiphytic lineages, we demonstrate that the evolution of epiphytism within *Peperomia* predates the diversification shift. An epiphytic-dependent diversification is only observed for the background phylogeny. An elevated frequency of life form transitions between epiphytes and terrestrials and thus evolutionary flexibility of life forms is uncovered to coincide with the diversification shift. The evolution of fruits showing dispersal related structures is key to diversification in the foreground region of the phylogeny and postdates the evolution of epiphytism. We conclude that the success of *Peperomia*, measured in species numbers, is likely the result of enhanced vertical and horizontal dispersal ability and life form flexibility but not the evolution of epiphytism itself.

## Introduction

Epiphytism evolved in various green plant lineages ranging from bryophytes and ferns to flowering plants (Gentry and Dodson, 1987). The majority of vascular epiphytes are found in the monocots and eudicots (75 and 13%, respectively) (Zotz, 2013). Epiphytism is generally hypothesized to enhance diversification, due to the large number of microhabitats and high niche fragmentation (Gentry and Dodson, 1987; Benzing, 1990). Orchids, Brome liaceae, and Gesneriaceae are well known examples of higher species richness in epiphytic flowering plant lineages, compared to terrestrial sister lineages (Gentry and Dodson, 1987; Gravendeel et al., 2004; Zotz, 2013; Givnish et al., 2015). This increased diversity has raised the question of factors shaping or contributing to the rate of diversification of epiphytic lineages. Especially in closed habitats such as tropical rainforests, the epiphytic life style provides potentially extended access to light compared to the terrestrial habitat. Epiphytism offers the possibility for occupying bark, branches and twigs that altogether provide a greater habitable surface compared to the ground level. Niche partitioning in climatic heterogeneous tree crowns furthermore is thought to maintain increased plant diversity (Gentry and Dodson, 1987; Benzing, 1990; Silvera et al., 2009). However, it is largely unclear whether morphological or physiological traits are a prerequisite for plants to become epiphytes or if character (trans) formations are a result of life form shifts and lead to diversification. Disentangling the temporal origin of traits and their impact on speciation or extinction rates is essential for understanding diversification and the underlying evolutionary processes.

With the rise of comparative evolutionary methods allowing for a trait’s influence on evolutionary processes, interferences on character state-dependent diversification can be directly inferred (e.g., Maddison et al., 2007; FitzJohn, 2012; Rabosky, 2014; Rabosky et al., 2014). Furthermore, methods for analyzing character state-independent diversification dynamics on phylogenies play a central role in phylogenetic research (Rabosky et al., 2007; Alfaro et al., 2009; Rabosky, 2014; Rabosky et al., 2014).

Recent studies confirmed the hypothesis of higher diversification rates in epiphytes compared to terrestrials within Bromeliaceae (Givnish et al., 2014) and Orchids (Givnish et al., 2015), and for epiphytes compared to generalists within ferns (Feldberg et al., 2014). Moreover, these new approaches reduce the biases in the interpretation of characters as possible key innovations (Ng and Smith, 2014). Key innovations represent novel characters, which are directly linked to increased diversification rates, either by reducing extinction or by accelerating speciation (Sanderson and Donoghue, 1994).

*Peperomia* (Piperaceae, Black Pepper relatives) is the only genus among the early diverging angiosperms (“basal angiosperms”, i.e., lineages which diverged prior to monocots and eudicots) where epiphytism evolved (Isnard et al., 2012). Among flowering plants, *Peperomia* comprises the largest number of epiphytic species in a genus apart from some orchid genera and *Tillandsia* (Bromeliaceae) (Zotz, 2013) and it is listed among the ten most species-rich flowering plant genera in general (1606 species) (Frodin, 2004; Samain et al., 2009, 2011; Mathieu et al., 2011; Frenzke et al., 2015). The genus is pantropically distributed with the highest diversity in the Neotropics (Wanke et al., 2006; Smith et al., 2008; Samain et al., 2011; Pino et al., 2012), where it is a notable component of the epiphytic (Barthlott et al., 2001; Krömer et al., 2007) and terrestrial (Mathieu et al., 2011; Samain et al., 2011) flora in a wide range of vegetation types. Molecular phylogenies of *Peperomia* revealed terrestrial clades to be successive sister to the remaining lineages in which epiphytism occurs at least frequently or is the predominant life form (Samain et al., 2009; Frenzke et al., 2015). The fruits (up to 3.5 mm in size) are small drupes consisting of a thin mesocarp, a stony endocarp and a hard testa (Johnson, 1900; Fisher, 1914; Burger, 1971). As many *Peperomia* species show sticky secretions on the fruit surface (Frenzke et al., 2016), epizoochorous dispersal has long been suggested by most (e.g., Dahlstedt, 1900; Ridley, 1930; Yuncker, 1958; Carlquist, 1967; Burger, 1971; Croat, 1978; Melcher et al., 2000), but not all authors (Bradley, 2002). Although information on *Peperomia* dispersing animals is sparse, birds are considered as vectors by Ridley (1930) and Carlquist (1967). The investigation of epizoochorous dispersal is fascinating as adhesion (including mechanical attachment mechanisms) is, with less than 5%, sparsely represented in plants. Even more particular is the adhesion by sticky substances (Sorensen, 1986). The majority of epiphytes (84%) propagate by tiny wind dispersed seeds called sporochores, followed by fruits showing winged or plumed seeds or adaptations to endozoochory (birds, bats) (Gentry and Dodson, 1987; Benzing, 2004). In contrast, terrestrial lineages frequently show more generalistic dispersal modes. Increasing dispersal ability and the colonization of new niches are thought to affect diversification as they act by altering gene flow (Ng and Smith, 2014). *Peperomia* is the only genus considered as epizoochorous throughout the literature on epiphyte dispersal (Yuncker, 1958; Madison, 1979; van der Pijl, 1982; Armesto and Rozzi, 1989; Hughes et al., 1994; Benzing, 2004) and to our knowledge the only epiphytic plant lineage developing sticky fruits. The species richness, the high number of epiphytes, as well as the fruit morphological modifications indicative for epizoochorous dispersal, make *Peperomia* a unique case to study life form evolution, fruit morphology, and diversification in a comparative manner.

We examined *Peperomia* fruits by cryo-scanning electron microscopy (cryo-SEM) to uncover dispersal-related traits. A molecular phylogeny of 114 *Peperomia* species covering the taxonomical and life form diversity of the genus is reconstructed. Life form is assigned to 1520 species. This framework serves as basis for state-dependent and state-independent diversification analyses, ancestral character state reconstructions, as well as tests for correlated evolution. We aim to test the overarching hypothesis that evolution of fruit morphological adaptations and transition from terrestrial to epiphytic life form fostered diversification in the species-rich genus *Peperomia*.

## Materials and Methods

One hundred fourteen *Peperomia* species were examined, representing the genus’ taxonomical, morphological and life form diversity (Frenzke et al., 2015). Plant material was collected in the field or taken from greenhouse collections of the Botanical Gardens of Ghent University (Belgium) and Dresden (Germany). Character scoring was completed with data from literature (e.g., Trelease and Yuncker, 1950; Yuncker, 1953, 1958, 1974; Samain et al., 2009). Successive sister lineages of *Peperomia* were used as outgroup (*Piper* and Saururaceae) (Wanke et al., 2007a,b; Samain et al., 2008; Naumann et al., 2011).

### Cryo-Scanning Electron Microscopy

We used Cryo-SEM to observe fruit micromorphology. Fruits were regarded as fully mature when they easily detach from the spadix upon slightest contact. Cryo-SEM protocols followed Frenzke et al. (2015).

### Phylogenetic Analyses

For the phylogenetic analysis based on the chloroplast *trn*K–*mat*K–*psb*A region, we sampled 114 *Peperomia* species. The selection was based on recently published molecular data (Frenzke et al., 2015), to which another 24 accessions were added. However, the molecular dataset includes less species compared to Frenzke et al. (2015), because precise information on fruit characters was not available for all species. Voucher and origin, as well as Genbank accession numbers of the 24 additional accessions are provided as supporting information (Supplementary Table S1). DNA extraction protocol, PCR settings, ingredients, and concentrations, as well as purification of products followed Frenzke et al. (2015). Sequences were obtained using Macrogen Inc. or a Beckman Coulter capillary lab sequencer. Sequence data were manually edited and aligned using PhyDE^®^ version 0.9971 (Müller et al., 2010), excluding regions of uncertain homology prior to analyses (Supplementary Table S2). A Maximum likelihood (ML) analysis was conducted using RAxML (Stamatakis et al., 2005) applying the GTR+Γ model and sampling 1000 rapid bootstrap replicates. The best fitting model was found using JMODELTEST (Guindon and Gascuel, 2003; Posada, 2008; Darriba et al., 2012). Bayesian Inference (BI) was performed using MRBAYES version 3.1.2 (Ronquist and Huelsenbeck, 2003) implementing four independent runs with four chains of 2,000,000 MCMC (Markov Chain Monte Carlo) generations each, saving every 200th tree. The first 300,000 generations of each run were discarded as burn-in as evaluated by TRACER (Rambaut and Drummond, 2009). FIGTREE version 1.4.0 (Rambaut, 2008) was used for tree editing.

**FIGURE 1:**
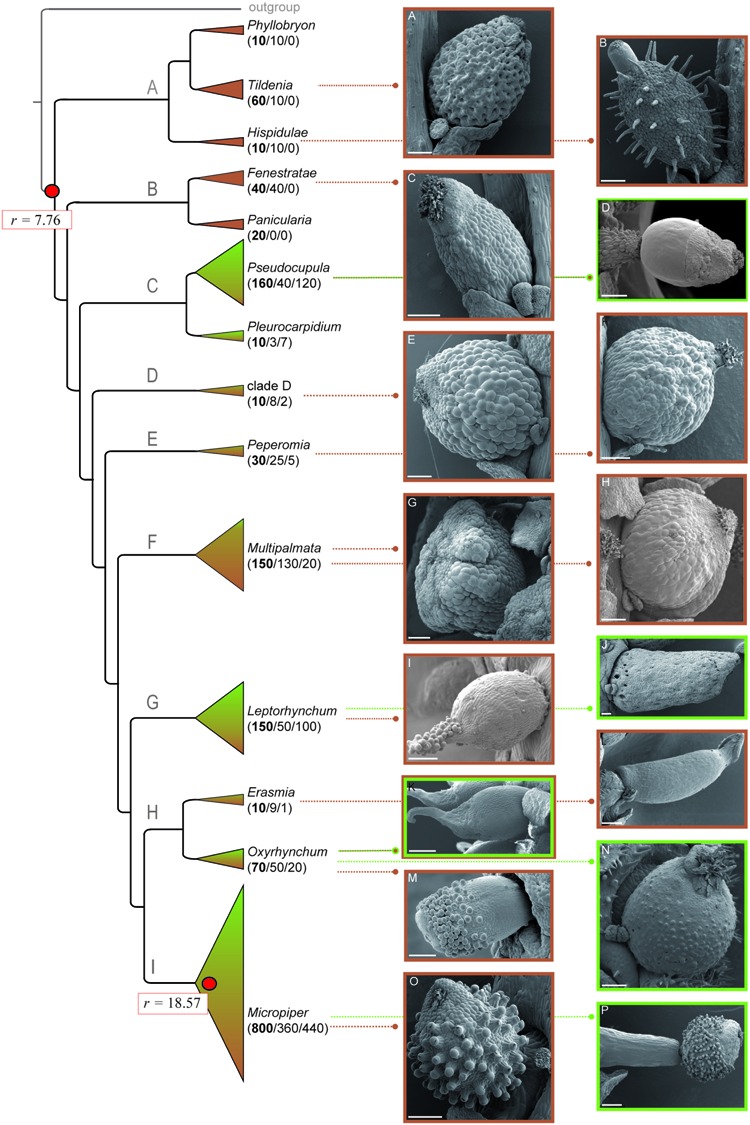
***Peperomia* fruit characters and life form distribution**. Pointed lines lead to SEM images of fruit phenotypes representative for each clade. Subgenera are named according to Frenzke et al. (2015). Color coding of picture frames and triangles of clades of the phylogenetic tree indicate the different life forms (brown-terrestrial; green-epiphytic). Shading of these clades is proportional to the respective species numbers being characterized by either terrestrial or epiphytic life form. Numbers in brackets refer to the estimated total number of species of each clade, the number of terrestrial species, and to the number of epiphytic species. The background rate for the entire tree, as well as the specific net diversification rate for the foreground clade are marked with red circles. **(A)** *P. bracteata*, **(B)** *P. hispidula*, **(C)** *P. dolabriformis*, **(D)** *P. dahlstedtii*, **(E)** *P. maypurensis*, **(F)** *P. pellucida*, **(G)** *P. procumbens*, **(H)** *P. arifolia*, **(I)** *P. species*, **(J)** *P. crassicaulis*, **(K)** *P. obtusifolia* var. *emarginata*, **(L)** *P. lancifolia*, **(M)** *P. pernambucensis*, **(N)** *P. hirta*, **(O)** *P. inaequalifolia*, **(P)** *P. bicolor*. Scale bars 200 μm.

The concatenated alignment of the trnK–matK–trnK–psbA dataset included 4418 characters from which 21 regions comprising 619 characters were excluded due to uncertain homology (Supplementary Table S2). Backbones of BI and ML topologies were highly congruent and supporting values for both methods are given on the ML tree (Supplementary Figure S1). The resulting majority rule (50%) consensus tree from BI analysis was used for our diversification study (**Figure 1**).

Further analyses required ultrametric phylogenetic trees. After pruning outgroup species from the consensus tree we run the ‘chronos’ function (Sanderson, 2002) of the package ‘ape‘ (Paradis et al., 2004) in R using RStudio 0.99.491 (R Studio Inc., Boston, MA, USA). We set the smoothing parameter to 0, allowing the full range of rate variation among branches. As there are no fossils recorded for *Peperomia*, we decided to set the tree height to 1 to avoid temporal bias (e.g., Granados Mendoza et al., 2015). All trees were subsequently rescaled assigning branch tips to time 0 and root to time 1.

### Diversification Shift Analysis

For the detection of heterogeneity in diversification rates and to test for credible rate shift configurations, we applied Bayesian analysis of macroevolutionary mixtures (BaMM, version 2.5.0). BaMM simulates posterior distributions of rate-shift configurations by ‘reversible jump’ Markov Chain Monte Carlo (rjMCMC) to account for rate variation through time and among lineages (Rabosky, 2014). BaMM can account for incomplete, non-random taxon sampling by allowing individual clades to have different sampling probabilities. We assigned the latest information on species richness to clades (**Figure 1**; Frenzke et al., 2015). The R function ‘BaMM priors’ was applied to get appropriate prior parameters based on the consensus tree. We choose a Poisson rate prior of 1.0, proposed by the default settings in BaMM as conservative approach. We run BaMM for ten chains of 5,000,000 generations, saving every 1000. Convergence and effective sample size were tested by applying the ‘R’ package CODA v. 0.18-1 (Plummer et al., 2006). We discarded 10% of the MCMC generations as burn-in prior to output analyses in R. The R package ‘BaMMtools’ (Rabosky et al., 2014) was used for post-run analysis of BaMM output files to identify the 95% credible set of shift configurations and to trace clade-specific diversification rates through times.

### Character Coding and Rationale

Fruit morphological characters and life form were scored (Supplementary Table S3). Life form was coded as terrestrial (0) or epiphytic (1), with geophytic and lithophytic considered as terrestrial (Zotz, 2013). We scored the general presence of fruit’s adhesiveness (manually tested) as absent (0) and present (1). It is, however, unclear if the fruit stickiness originates from anatomically homologs structures. Fruit adhesiveness is caused by epidermal structures, which may be secretive papillate cells (*Peperomia* subgenera *Micropiper*, *Oxyrhynchum*, *Leptorhynchum*), or the so-called pseudocupula (*Peperomia* subg. *Pseudocupula*). The latter was reported as an epidermal structure covering the fruit base (Dahlstedt, 1900). In addition, punctual sticky secretion is observed as an intermediate state in some species of *Peperomia* subg. *Oxyrhynchum*. In those cases, stickiness could be verified, but the secreting structures were neither pseudocupula nor papillate cells. Fruit trichomes of *P. hispidula* and *P. hispiduliformis* were not regarded as adhesive because they are not glandular (Martínez-Colín et al., 2006). The pseudopedicel represents a stalk-like outgrowth of the rachis and has been regarded as a dispersal-related structure that develops during fruit maturation (Dahlstedt, 1900; Yuncker, 1958). It was coded as absent (0) or present (1). The fruit apex is potentially relevant for dispersal as it forms hook-shaped appendages in some species and was coded as being absent (0) or present (1).

For the analyses of fruit character evolution we pruned outgroup species, as character states were not applicable. Drupes of the genus *Piper* differ notably in size, color and amount of fleshy mesocarp and show no obvious structures for mechanical attachment and external dispersal (Yuncker, 1958) (Rauh, 1950). The analysis of life form is based on 1520 *Peperomia* species and additionally includes outgroup lineages (Isnard et al., 2012).

### Binary State Speciation and Extinction Analyses

We used BiSSE (Maddison et al., 2007) as implemented in the R package ‘diversitree’ (FitzJohn, 2012) to estimate evolutionary parameters. BiSSE simultaneously estimates diversification and transition rates without requiring ancestral state reconstructions (ASRs). The BiSSE model assumes that speciation and extinction follow a birth–death process, where rates depend on a certain character state. It thus adds character-dependent parameters to the commonly used Markov models of character evolution (Mk models; Pagel, 1994; O’Meara, 2012). All BiSSE calculations were performed on the consensus tree obtained by BI. We applied the ‘skeletal tree’ approach with a sampling frequency of 0.1 for the fruit morphological characters. For some species, fruit characters could not be studied and information on fruit characters in the literature is often vague. We assume our sampling being representative as species of every clade of the phylogeny were included in the analysis. Within clades a random subset of one to 20 species was sampled in relation to clade size. Information on life forms of all known *Peperomia* species was included by applying the ‘unresolved tree approach’ for this character where life form distributions were assigned to terminal clades (**Figure 1**, numbers in brackets). Twelve different evolutionary models for life form and fruit functional characters were compared, testing character-associated parameters. The full BiSSE model includes six parameters, where speciation (λ), extinction (μ), and transition (*q*) rates are calculated for each of the two character states (0, 1). We compared the full model (λ_0_, λ_1_, μ_0_, μ_1_, *q*_01_, *q*_10_) to constrained models with speciation, extinction, and transition rates subsequently set to equal or zero (λ_0_ = 0; λ_1_ = 0; λ_0_ = λ_1;_ μ_0_ = 0; μ_1_ = 0; μ_0_ = μ_1,_ *q*_01_ = 0; *q*_10_ = 0; *q*_01_ = *q*_10_). We tested the Mk2 model as alternative hypothesis to explain character distributions independent from state-specific speciation and extinction. The Mk2 model (4-parameter model) was treated as a constrained model of the full model with speciation and extinction rates set equal for both states and transition rates varied freely (λ_0_ = λ_1_, μ_0_ = μ_1_). To account for heterogeneity in diversification rates related to life form and fruit character evolution, we furthermore applied the function ‘bisse.split’. ‘Bisse.split’ uses an expanded 12-parameter BiSSE model where parameter sets are estimated independently for partitions of the phylogeny. We chose to split the phylogeny at the point that is identified by BaMM as the most probable shift of the 95% credible set of shift configurations (*P* = 0.65, Supplementary Figure S2A). Hence, parameters were estimated separately for the background (λ_0.1_, λ_1.1_, μ_0.1_, μ_1.1_, *q*_01.1_, *q*_10.1_) and the foreground partition (λ_0.2_, λ_1.2_, μ_0.2_, μ_1.2_, *q*_01.2_, *q*_10.2_). In a first step, model parameters were optimized with ML searches on our consensus tree obtained by BI. To test which model explains our data best, and thus how life form and dispersal related fruit characters have influenced diversification we compared the log likelihoods (lnLs) of all models using the AIC (Maddison et al., 2007) and related Akaike weights (*w*_i_) (Burnham and Anderson, 2002).

To account for parameter uncertainty we performed Bayesian MCMC using slice sampling (Neal, 2003) under the best fitting model. An exponential prior with rate 1/(2*r*) was used for MCMC simulations. *r* is the character-independent diversification rate obtained by BiSSE ML (Maximum Likelihood) estimation. A preliminary set of 1,000 MCMC steps was run to obtain *a priori* estimates for the tuning parameter *w* (0.95 density of PP) (Neal, 2003). Final MCMC chains (with *w* specified) were run for 20,000 iterations to obtain posterior distributions of evolutionary parameters. Although chains converged rapidly, a burn-in fraction of 25% MCMC steps was discarded (conservative approach). Post burn-in MCMC samples were tested for effective sample size above 200 using the MCMC output analysis and diagnosis package ‘coda‘ (Plummer et al., 2006) implemented in R. 95% Credibility Intervals (CIs) of posterior distributions are provided for each parameter set. We summed proportions of post burn-in samples with higher rates for state 1 compared to state 0 and treated them as PP. We considered PP ≥ 0.95 as support for significant higher rates.

Rabosky and Goldberg (2015) recently showed that if a tree evolves under a heterogeneous branching process that is completely independent from the evolution of the character to be investigated; SSE models [state speciation and extinction models; see Rabosky and Goldberg (2015)] will, in many cases, return high support for a model of trait-dependent diversification. By applying BaMM as cross-validation to test for model violation and by applying a partitioned SSE model to account for diversification rate heterogeneity in our phylogeny, we address for possible inadequacy reported by Rabosky and Goldberg (2015). ASR was performed under the best fitting BiSSE (Binary State Speciation and Extinction) model to account for the influence of life forms and fruit character states on the diversification of *Peperomia*. We applied the BiSSE marginal ASR as implemented in the R-package ‘diversitree’ (FitzJohn, 2012). To integrate parameter uncertainty in ancestral character state reconstructions we run 1,000 MCMC iterations. The same priors as for BiSSE MCMC analysis were applied to start ASR-MCMC chains. Mean values of proportional likelihoods were calculated after discarding a burn-in of 25% from the MCMC samples and ancestral state probabilities were mapped onto main nodes of the consensus tree with a particular state being most likely at a significance ratio of 0.86 (7.4:1) or above (Edwards, 1972; Schluter et al., 1997).

### Tests for Correlated Evolution

Correlated evolution among fruit characters and life form was tested using Pagel’s correlation analysis (Pagel, 1994) implemented in the Correl Package version 0.1 of Mesquite (Midford and Maddison, 2006). We run this analysis as an exploratory analysis without specifying *a priori* assumptions on the evolutionary association for all pairwise combinations. Pagel’s approach uses the LRT statistic to discriminate between two models. One model assumes for correlated state changes of the two tested characters and consequently applies eight parameters. The second model represents a constrained version of the eight-parameter model and describes the transition between character states independently from transitions of the second character using four parameters. We run Pagel’s test on a subset of ten BI trees applying 30 ML iterations. Simulations were computationally expensive and did not affect *P*-values. We therefore decided to run 1000 MCMC iterations on only one tree of the subset for each character combination. For comparison of model fitting, AIC and *w*_i_ were calculated as described above. Accessions with unknown or missing character states were excluded prior to analyses.

## Results

A-well resolved and supported phylogeny based on 4118 bp alignment characters resulted in nine main lineages (**Figures 1 A–I**; Supplementary Figure S1), corresponding to subgenera and unnamed clades (**Figure 1**, Supplementary Figure S1, Frenzke et al., 2015). Lineages A and B comprise exclusively terrestrial species, lacking fruit characteristics such as pseudopedicel, sticky secretions, and beaks (**Figures 1A–C**). Lineage C includes 18% (127 species) of all epiphytes. Fruits of *Peperomia* subg. *Pseudocupula* are characterized by sticky secretions on the fruit surface, the so-called pseudocupula (**Figure 1D**) (Samain et al., 2009; Frenzke et al., 2015). More than 50% of the sampled *Peperomia* subg. *Pseudocupula* species exhibit a pseudopedicel, but rarely show apical beaks. The species-poor *Peperomia* subg. *Pleurocarpidium* being sister to *Peperomia* subg. *Pseudocupula* shows none of these structures. The unnamed clade D and *Peperomia* subg. *Peperomia* harbor predominantly terrestrial species and lack sticky secretions, pseudopedicel, as well as beaks (**Figures 1E,F**). *Peperomia* subg. *Multipalmata* as sister to the remaining *Peperomia* lineages is a species-rich group comprising 18% of all terrestrial *Peperomia* species. Diversity of fruit shapes is high in this subgenus but no means for external dispersal are observed (**Figures 1G,H**). The species-rich *Peperomia* subg. *Leptorhynchum* comprises one third terrestrial and two thirds epiphytic species. Species of this clade are morphological diverse with respect to fruit shapes and structures. They show sticky secretions, as well as beaks or hooks of varying length (**Figures 1I,J**). The fruits show a sessile or subsessile attachment without pseudopedicel formation. Lineage H includes the two smaller subgenera *Erasmia* and *Oxyrhynchum* (**Figure 1**). The mainly terrestrial *Peperomia* subg. *Erasmia* is characterized by elongated fruits with beaks and pseudopedicel formation, but no sticky secretions are observed (**Figure 1L**). *Peperomia* subg. *Oxyrhynchum* harbors morphologically diverse fruits with beaks or hooks and sticky secretions (**Figures 1K–N**). The most species-rich *Peperomia* subg. *Micropiper* (800 species) includes similar proportions of terrestrial and epiphytic species (**Figure 1**). In contrast to most other *Peperomia* lineages, all fruits of subg. *Micropiper* are characterized by sticky papillae and a pseudopedicel, and do often show a beaked apex as well (**Figures 1O,P**).

### Diversification within *Peperomia*

**FIGURE 2:**
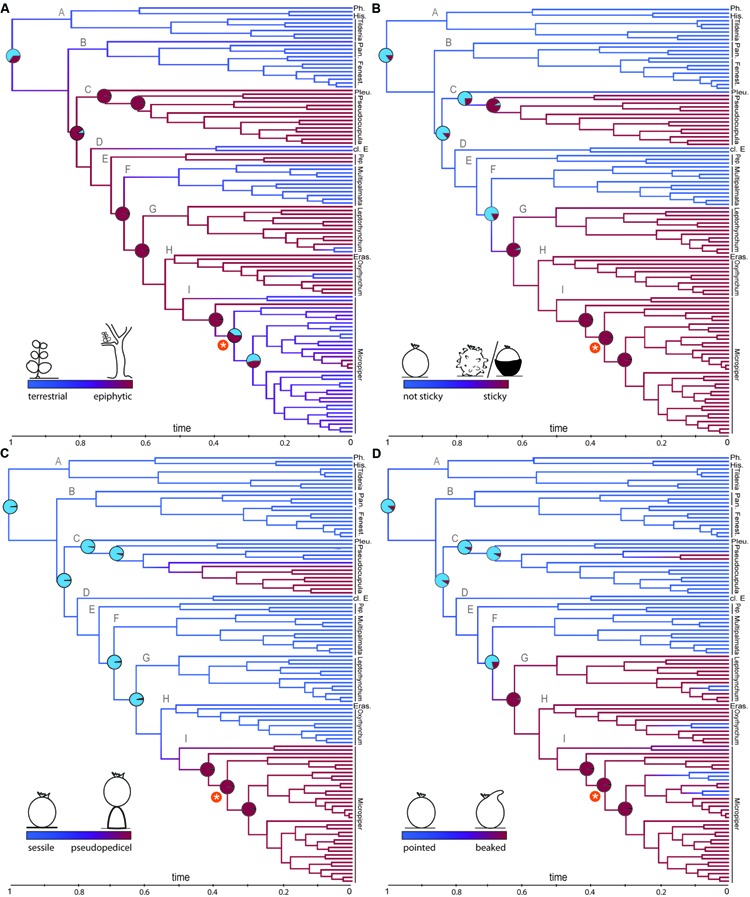
**Reconstruction of fruit traits and life form**. Ancestral state reconstruction (ASR) using the BiSSE model is shown on the majority rule (50%) consensus tree obtained from BI analysis. **(A)** Epiphytism evolved before the diversification shift, **(B–D)** Studied fruit characters each originated twice along the phylogeny. Branch colors refer to state probabilities. Pie charts illustrate character state probabilities for main nodes. The asterisk marks the diversification shift within subg. *Micropiper*.

Bayesian analysis of macroevolutionary mixture Markov Chain Monte Carlo runs converged rapidly and our burn-in fraction of 0.1 was proved to be sufficient by CODA MCMC output analysis. Effective sample sizes were much greater than 200 with 2158 for the number of shift events along the phylogeny and 1525 for the lnL. ‘BaMMtools’ reveal our *Peperomia* phylogeny to violate a single regime birth-death model. The 95% credible set of shift configurations indicates rate heterogeneity within *Peperomia*, due to a single diversification shift (Supplementary Figure S2). The mean net diversification rate of the phylogeny is estimated with *r* = 7.76 (Supplementary Figure S2B). Frequently sampled shifts of increased net diversification rate are found between time 0.28 and 0.40 located within subgenus *Micropiper* (**Figure 2**, Supplementary Figure S2B). The average net diversification rate beyond this shift (*r* = 18.57) exceeds twice the mean background rate for the entire tree. Clade specific diversification rates are estimated with mean values between 6.29 and 7.07 for all *Peperomia* subgenera except for subgenus *Micropiper* (*r* = 15.91; Supplementary Figures S2A,B).

### Character-Dependent Diversification

Our character-dependent diversification analyses shows that the full BiSSE split model, which estimates rates of speciation, extinction and character changes independently for the foreground and the background phylogeny, provides significant better fit for all examined traits than all other models (Supplementary Table S4).

**FIGURE 3:**
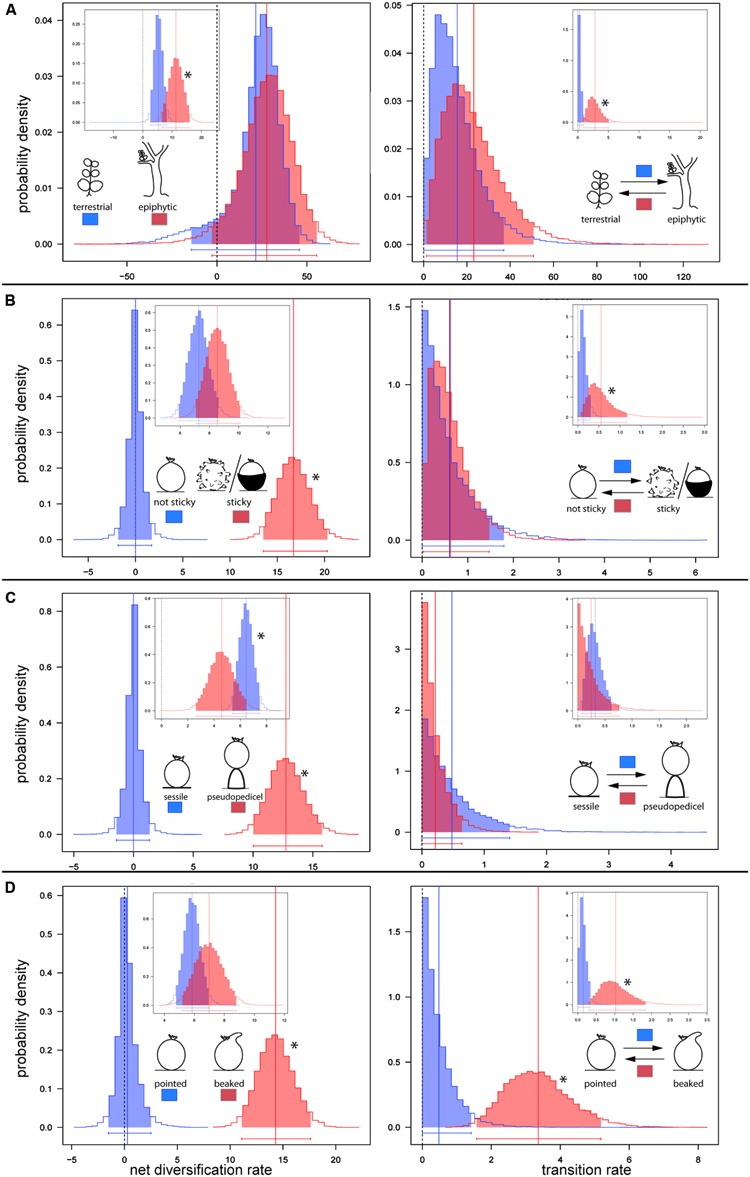
**Posterior distributions of diversification rate (left) and character transition rate (right)**. Rates were estimated by BiSSE MCMC under the 12-parameter full BiSSE split model. Main profile plots show foreground parameter estimates. Inbox plots show respective estimations for the background phylogeny. Rate estimations are shown for **(A)** terrestrial and epiphytic *Peperomia* lineages; **(B)** for lineages lacking and those possessing sticky fruits; **(C)** for lineages without pseudopedicel and with pseudopedicel formation, and **(D)** for lineages showing pointed fruits and those with beaked fruits. ^∗^ marks significantly higher rates for one state over another.

Our BiSSE MCMC analyses reveal epiphytic lineages in the background region to diversify twice as fast as the terrestrial ones (**Table 1**; **Figure 3A**). Transitions from epiphytes to terrestrials generally occur at higher rates than in the reverse direction, however, being significant in the background lineages only (**Tables 1** and **2**; **Figure 3A**). Diversification and transition rates for both epiphytes and terrestrials increase considerably after the diversification shift (**Table 1**; **Figure 3A**). However, no differences are identified between both character states in the foreground clade as indicated by largely overlapping 95% credible intervals (CIs; **Tables 1** and **2**; **Figure 3A**).

**Table 1:** Means of post burn-in posterior estimates of speciation, extinction, rate of character change, and diversification obtained by BiSSE MCMC under the 12-parameter full BiSSE split model.

		λ_0_	λ_1_	μ_0_	μ_1_	*q*_01_	*q*_10_	*r*_0_ = λ_0_ -μ_0_	*r*_1_ = λ_1_ -μ_1_
Life form	Background	20.82 (10.13; 33.30)	29.64 (14.47; 49.36)	15.47 (3.35; 29.82)	18.27 (0.03; 39.76)	0.34 (0.00; 0.96)	2.81 (0.87; 5.04)	5.35 (2.60; 8.21)	11.37 (6.60; 16.01)
	Foreground	32.86 (0.86; 52.82)	38.69 (9.33; 67.64)	11.31 (0.00; 32.51)	11.02 (0.00; 31.63)	15.56 (0.01; 37.05)	23.18 (0.97; 50.39)	21.55 (-14.19; 45.91)	27.67 (-2.63; 55.62)
Stickiness	Background	7.85 (6.35; 9.38)	8.98 (7.39; 10.63)	0.57 (0.00; 1.64)	0.44 (0.00; 1.30)	0.13 (0.00; 0.30)	0.55 (0.08; 1.17)	7.28 (5.94; 8.63)	8.54 (7.08; 10.10)
	Foreground	0.61 (0.00; 1.77)	17.11 (13.87; 20.57)	0.59 (0.00; 1.75)	0.39 (0.00; 1.18)	0.60 (0.00; 1.79)	0.61 (0.01; 1.46)	0.01 (-1.83; 1.71)	16.72 (13.51; 20.29)
Pseudopedicel	Background	6. 86 (5. 73; 8.01)	4.89 (2.99; 6.84)	0.40 (0.00; 1.17)	0.30 (0.00; 0.91)	0.32 (0.07; 0.63)	0.25 (0.00; 0.77)	6.45 (5.41; 7.50)	4.59 (2.62; 6.45)
	Foreground	0.46 (0.00; 1.38)	13.05 (10.17; 15.92)	0.46 (0.00; 1.38)	0.29 (0.00; 0.89)	0.48 (0.00; 1.41)	0.21 (0.00; 0.64)	0.00 (-1.38; 1.40)	12.75 (9.98; 15.74)
Style protuberance	Background	6.32 (5.15; 7.58)	7.36 (5.53; 9.20)	0.46 (0.00; 1.33)	0.37 (0.00; 1.12)	0.15 (0.00; 0.33)	1.03 (0.31; 1.83)	5.86 (4.78; 6.95)	6.98 (5.19; 8.79)


	Foreground	0.79 (0.00; 2.40)	14.67 (11.48; 18.01)	0.51 (0.00; 1. 55)	0.36 (0.00; 1.10)	0.48 (0.00; 1.42)	3.36 (1.60; 5.20)	0.28 (-1.50; 2.52)	14.30 (11.23; 17.75)

*Parameters were estimated independently for two regions of the phylogeny. Lower and upper bound values of the 95% credibility interval are given in parentheses. Subscript numbers refer to terrestrial (0) and epiphytic (1) life form, as to the absence (0) or presence (1) of fruit characters, respectively. All parameters refer to a total tree depth of 1*.

Our results inferred generally higher diversification rates for species with sticky fruits (**Table 1**; **Figure 3B**). For this character state foreground diversification rates are found to be significantly higher (no overlapping CIs) (**Table 2**; **Figure 3B**). Transition rates in both directions are found to be generally low in the background phylogeny, however, with significantly lower probability for the gain of fruit stickiness (**Table 1**; **Figure 3B**).

**Table 2:** Differences of transition and diversification rates inferred from MCMC sample proportions.

	Life form		Stickiness		Pseudopedicel		Beak	
	Background	Foreground	Background	Foreground	Background	Foreground	Background	Foreground
PP (*q*_10_ > *q*_01_)	0.99^∗^	0.73	0.95^∗^	0.56	0.30	0.31	>0.99^∗^	>0.99^∗^
PP (*r*_1_ *> r*_0_)	0.98^∗^	0.57	0.88	>0.99^∗^	0.05	>0.99^∗^	0.84	>0.99^∗^

*Proportions were treated as posterior probabilities. 0.05 ≥ PP ≥ 0.95 is considered as support for significant different rates in epiphytes versus terrestrials; in lineages exhibiting sticky fruits, a pseudopedicel or beak compared to those lacking these fruit structures. Subscript numbers refer to terrestrial (0) and epiphytic (1), as to the absence (0) or presence (1) of fruit characters. ^∗^ marks significant differences*.

Markov Chain Monte Carlo analyses under the full BiSSE split model show that species with sessile fruits diversify significantly faster than those possessing a pseudopedicel in the background phylogeny. However, the reverse is found for the foreground clade where pseudopedicellate species diversify at significantly higher rates (**Table 1**; **Figure 3C**). Largely overlapping posterior distributions in both background and foreground partitions indicate no differences in transition rates between both character states (**Table 2**; **Figure 3C**). Diversification rates of species with a fruit beak are generally higher than those lacking this structure, but this is only significant in the foreground partition (**Tables 1** and **2**; **Figure 3D**). The evolution of the beak was recovered with significantly higher loss than gain rates across the entire phylogeny (**Tables 1** and **2**; **Figure 3D**).

### Ancestral Character States

Ancestral character state reconstructions under the full BiSSE split model inferred the most recent common ancestor (MRCA) of *Peperomia* to be most likely terrestrial (*P* = 0.73, **Figure 2A**). An epiphytic MRCA is reconstructed for the node of lineage C to all remaining lineages. At the shift of increased diversification rate an ambiguous reconstruction with respect to terrestrial/epiphytic is reconstructed (**Figure 2A**). Within the foreground clade, frequent reversals to a terrestrial life form are observed (**Figure 2A**).

The MRCA of the genus is reconstructed to lack sticky fruits (**Figure 2B**; *P* = 0.87). Sticky fruits are recovered with two independent origins, predating the diversification shift (**Figure 2B**). Reversals to fruits lacking sticky structures were not recovered.

A sessile attachment of fruits is reconstructed as ancestral state for the MRCA of *Peperomia* (*P* = 0.97; **Figure 2C**), while the pseudopedicel evolved twice independently, once within subg. *Pseudocupula* and once within subg. *Micropiper* (**Figure 2C**). Reverse transitions to fruits without pedicels are not discovered.

Pointed fruits are the most likely ancestral state for *Peperomia* (*P* = 0.88, **Figure 2D**) and an apical beak-shaped protuberance originated at least twice. Frequent reversals to fruits lacking the apical beak are observed within the subgenera *Leptorhynchum*, *Oxyrhynchum* and *Micropiper* (**Figure 2D**).

### Correlated Evolution

The dependent model (four-parameter) is preferred over the model of independent evolution (eight-parameter) due to a higher likelihood and higher *w*_i_ for all tested character combinations. Correlated evolution of life form and fruit stickiness is statistically supported (**Table 3**, *w*_i_ > 0.99) in all trees. In addition, the dependent parameter model favored the explanation of epiphytic evolution in association with fruit beaks in 90% of the analyzed trees (**Table 3**). Testing all fruit character combinations, only fruit beak and pseudopedicel evolved in correlation (**Table 3**, nine trees, *w*_i_ > 0.96). Evolutionary changes in life form are unrelated to pseudopedicel evolution (**Table 3**).

**Table 3:** Means of log likelihood (lnL) and Akaike information criterion (AIC) of the two tested models for all possible pairs of characters.

Character combination	4-parameter model		8-parameter model		ΔAIC	*w*_i_	# trees supporting 8-par. Model
	lnL	AIC	lnL	AIC			
epiphytism / fruit stickiness	-84.18	176.37	-71.48	158.96	17.41	>0.99	10/10
epiphytism / pseudopedicel	-97.24	202.47	-90.25	196.49	5.98	>0.97	3/10
epiphytism / fruit beak	-114.73	237.47	-106.09	228.18	9.92	>0.98	9/10
fruit stickiness / pseudopedicel	-63.20	134.41	-57.00	130.00	4.41	>0.96	2/10
fruit stickiness / fruit beak	-81.29	170.58	-73.79	163.59	6.99	>0.97	6/10
pseudopedicel / fruit beak	-93.76	195.51	-85.25	186.49	9.02	>0.96	9/10

*The mean difference between the models is given as ΔAIC. The last column presents the number of trees from the random subset of ten trees where the dependent 8-parameter model was significantly supported over the independent 4-parameter model by Akaike weights (*w*_i_) above 0.95*.

## Discussion

Here we present the first study on diversification of the genus *Peperomia* that belongs to the top 10 most species-rich genera of angiosperms (Frodin, 2004). Former studies addressing the evolution of the genus mainly focused on systematics (e.g., Samain et al., 2007, 2011; Mathieu et al., 2008; Frenzke et al., 2015), biogeography (Symmank et al., 2008, 2011), relationships among clades (Wanke et al., 2006; Samain et al., 2009) or life and growth forms in a broader context (Isnard et al., 2012). These studies form the basis to combine knowledge on characteristic structures and molecular phylogenetics in comparative analyses to figure out whether and how life forms and fruit structures influence diversification within *Peperomia*.

We demonstrate that diversification within *Peperomia* is not homogenous across its evolution and more complex than initially thought. Given that the full BiSSE split model best fits our data, we confirm that life form and fruit characters did not evolve in a constant manner throughout *Peperomia*. Although the shift to epiphytism was likely a driver of diversification, the increase in diversification of the most species-rich subgenus rather correlates with fruit characters. We rather hypothesize that evolutionary flexibility of life forms coincides with the diversification shift.

As already indicated in the methods section, SSE approaches may infer associations of neutral traits and diversification especially if only a single diversification shift is discovered (Rabosky and Goldberg, 2015) and thus results have to be interpreted carefully. However, we choose a cross-validation by applying both BaMM and BiSSE approaches to reduce false positive results. We are furthermore aware that our study provides a first glimpse because diversification might be influenced by a complex interplay of ecological and morphological features. Additionally, conclusions on extinction rates should be drawn carefully in the absence of a fossil record (Rabosky, 2010).

### Life Form and Diversification

Epiphytism is generally regarded as enhancer of diversification (Gentry and Dodson, 1987; Benzing, 2004). For epiphytic bromeliad subfamilies, Givnish et al. (2014) uncovered high rates of net diversification. A similar finding was uncovered for orchids, where the epiphytic habit appears to have accelerated net diversification rates (Givnish et al., 2015). In *Peperomia*, the epiphytic life form was driving diversification before the rate shift (**Figure 3A**). Life form associated rate differences disappear after the diversification shift, where speciation and extinction rates of epiphytes and terrestrials are found to be similar. Hence, epiphytism is not linked to the diversification shift although our analyses indicate a gradual evolution to epiphytism in background lineages. After the diversification shift we observe a high frequency of reversals and equal transition rates between epiphytes and terrestrials. This is in contrast to other flowering plant lineages where reversals from an epiphytic to terrestrial life form are uncommon (Crayn et al., 2004; Gravendeel et al., 2004; Givnish et al., 2014). Although epiphytism has been shown to be a result of a suite of key innovations (Gravendeel et al., 2004), we rather hypothesize that the terrestrial ancestor of *Peperomia* already showed traits potentially facilitating epiphytic life such as succulence or CAM metabolism.

### Fruit Characters and Diversification

In addition to the evolution of epiphytism, *Peperomia* is also the only lineage among the Magnoliids that includes species with sticky fruits. Diversity of fruit morphology such as sticky means, appendages like hooks or beaks, and/or the pseudopedicel are characteristic for the majority of *Peperomia* species. Fruits lacking these structures are ancestral in *Peperomia* and mark the early diverging clades *Phyllobryon*, *Hispidulae, Tildenia, Panicularia*, and *Fenestratae*, as well as the later diverging lineages *Multipalmata, Peperomia*, and clade D (**Figure 2**). It is noteworthy that fruit stickiness and the pseudopedicel originated repeatedly and were not lost anymore in contrast to epiphytism. The irreversibility of fruit morphological changes including the development of new structures or fundamental reorganization seems to be a common finding across angiosperms (Beaulieu and Donoghue, 2013; Givnish et al., 2014). According to our reconstructions all studied fruit traits originated in the same clades (clade C and sister lineage of clade F, **Figure 2**). Most of the species of *Peperomia* subg. *Micropiper* show sticky secretions, apical beaks, and develop a pseudopedicel. Likewise, we find the same characters originating within *Peperomia* subg. *Pseudocupula*. Hence, these traits represent interesting cases of apparent convergence and are seemingly the result of similar adaptive forces acting on *Peperomia* fruit evolution within these two groups. According to BiSSE analyses (**Figure 3**), all examined fruit characters are positively associated with diversification across *Peperomia* evolution in the foreground clade. Although environmental changes involve the creation of key opportunities, plants must be able to access the new niche space. Furthermore, increased ability to disperse is prone to considerably affect diversification (Ng and Smith, 2014) and recent comparative studies provide evidence for fruit types and structures to influence diversification (Beaulieu and Donoghue, 2013). In *Peperomia*, the fruit type is conserved, i.e., the drupe, and the fruit structures studied here are suitable means for efficient dispersal. Hence, we are confident that the evolution of the examined fruit traits reflects the transition from abiotic to biotic dispersal within *Peperomia* and increased diversification in the foreground clade.

Our correlation analysis reveals that directional changes to epiphytism correlate with transitions to sticky fruits. However, it is important to note that according to our reconstructions, fruit traits have not been a prerequisite for the evolution of epiphytism. Moreover, we provide evidence that the ability to stick to potential vectors and the substrate has been beneficial for epiphytic as well as secondary terrestrial species.

### Evolutionary Consequences for Dispersal

*Peperomia* species with different fruit structures can be assumed to be “adhesively dispersed” according to dispersal type definitions (Willson and Traveset, 1990). Sorensen (1986) and Bremer and Eriksson (1992) stated that few structural changes may be required to change, e.g., from abiotic to biotic dispersal. The general appearance of *Peperomia* fruits with a stony endocarp and the lack of visual attractiveness furthermore support the assumption of undirected, passive dispersal (van der Pijl, 1982; Dahlstedt, 1900; Kubitzki et al., 1993) by, e.g., birds (Ridley, 1930; Carlquist, 1967).

Fruits with the ability to attach to vectors are often considered key to long distance dispersal (LDD), explaining isolated populations (Sorensen, 1986; Baldwin and Wagner, 2010). The positive effect of fruit stickiness on horizontal and vertical dispersability was already stated for *Peperomia* by Burger in 1971: “[...] This character which allows peperomias to reach high tree-tops has resulted in many geographically widespread species”. On the one hand, the only two subgenera *Micropiper* and *Pseudocupula* showing pantropical distribution that also includes remote islands provide support that *Peperomia* species with sticky fruits have access to an extended geographic range. The interoceanic long-distance dispersal is one of the longest ever reported for angiosperms (Valdebenito et al., 1990, 1992). On the other hand, the lack of functional adaptations to LDD has previously been reported as a reason for the restricted distribution of clades such as *Tildenia* (Symmank et al., 2011).

## Author Contributions

LF, M-SS, and SW conceived and designed the study. LF performed the analyses. PG, M-SS, CN, and SW contributed to data acquisition and interpretation. LF wrote the manuscript with the help of M-SS and SW. All authors contributed to the discussion, revised, and approved the final manuscript.

## Conflict of Interest Statement

The authors declare that the research was conducted in the absence of any commercial or financial relationships that could be construed as a potential conflict of interest.
