# Supplementary material for: Evolution of Epiphytism and Fruit Traits Act Unevenly on the Diversification of the Species-Rich Genus Peperomia (Piperaceae)
Source: Front Plant Sci. 2016 Aug 9;7:1145. doi: 10.3389/fpls.2016.01145 (PMC4977276; doi:10.3389/fpls.2016.01145)
Supplement: Supplementary file 1 [file Data_Sheet_1.PDF]

Supplementary Material

Evolution of Epiphytism and Fruit Traits Act Unevenly on the Diversification of the Species-Rich Genus *Peperomia* (Piperaceae)

Lena Frenzke[1], Paul Goetghebeur[2], Christoph Neinhuis[1], Marie-Stéphanie Samain[2, 3] Stefan Wanke[1*]

[*]Correspondence:

Stefan Wanke

stefan.wanke@tu-dresden.de

Fig. S1 Full phylogenetic hypothesis obtained by ML with ML bootstrap values plotted above and Posterior Probability (PP) values from Bayesian Inference plotted below branches.

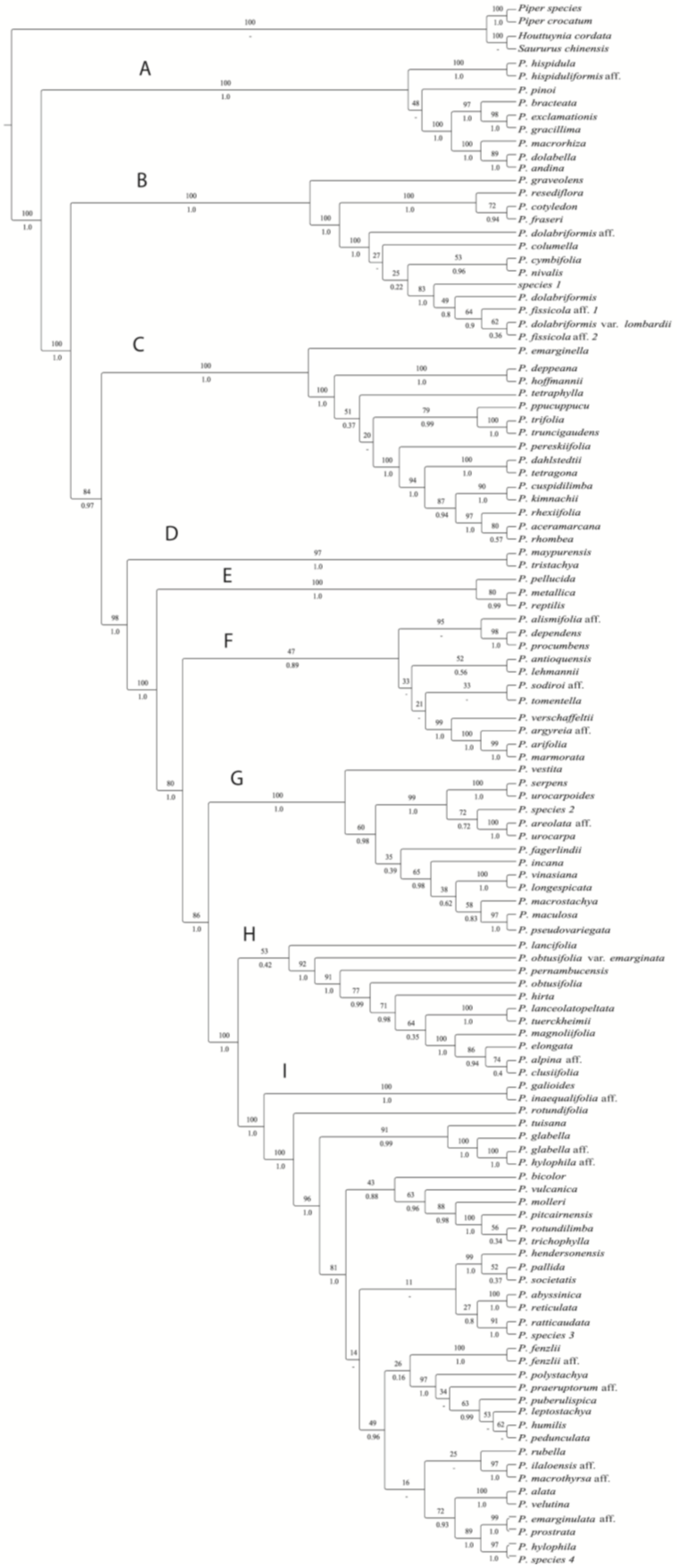

Fig. S2 BaMM output analysis. A) Phylorate plots of the 95% credible set of shift configurations, B) Lineage through time plots of net diversification rates for the background phylogeny and the *Peperomia* subg. *Micropiper*.

A

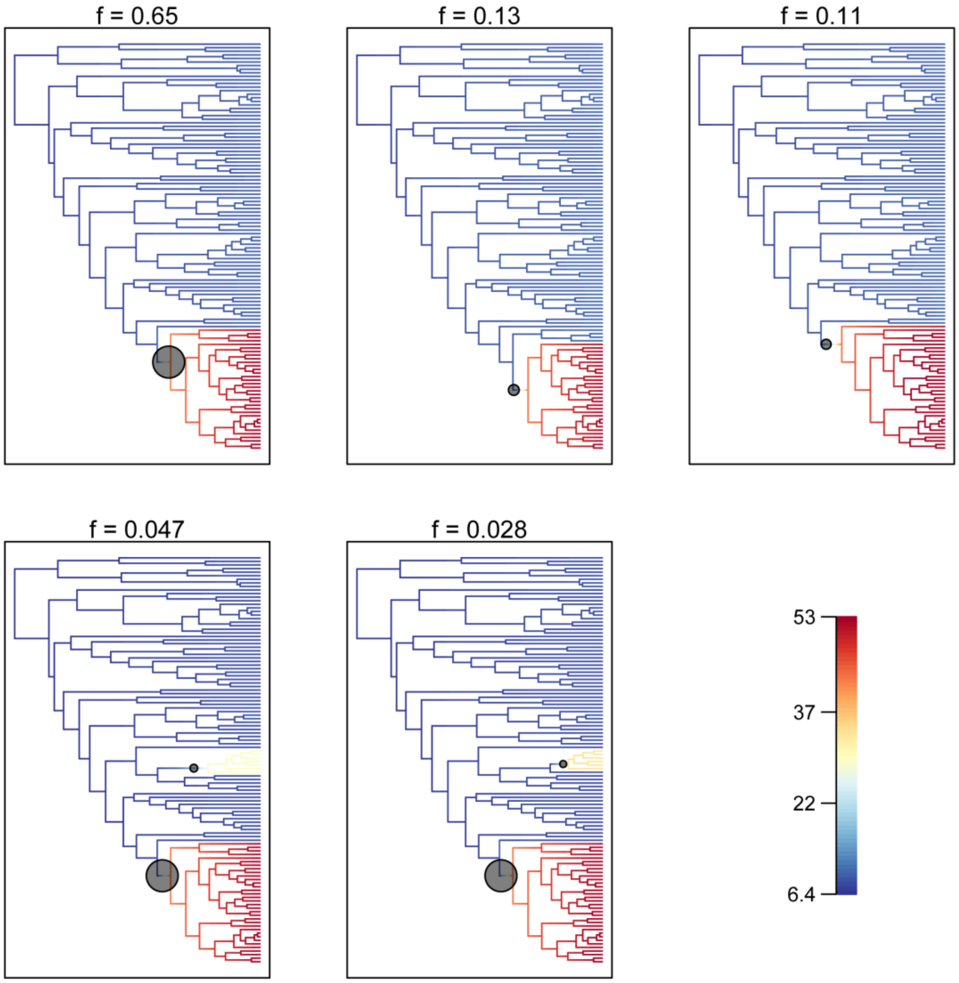

B

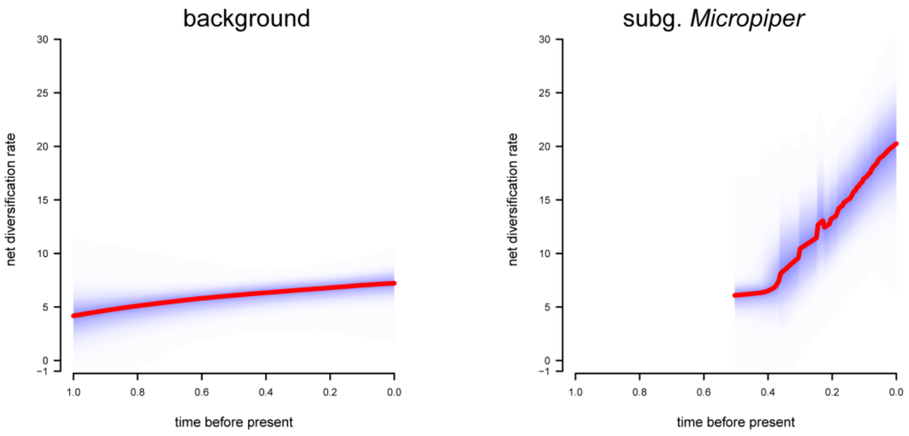

Table S1 Taxa, voucher information, GenBank accession numbers as well as field or garden origin are given for accessions which were added to the molecular sampling of Peperomia. Abbreviations: AAU - Herbarium of Aarhus University, DR – Herbarium of the Botanic Garden Dresden, GENT - Herbarium of the Ghent University, LPB - Herbario Nacional de Bolivia, MEXU - Universidad Nacional Autónoma de México, USM - Universidad Nacional Mayor de San Marcos.

species	lab no.	voucher and origin of molecular samples	Genbank accession
P. alismifolia **Presl aff.**	Pe765	*Samain et al.* 2010-193 (GENT, USM); BG Gent, 2011 0027	KX451148
P. alpina **(Sw.) A.Dietr. aff.**	Pe905	*Frenzke* 005 (DR); BG Gent, 2006 1279	KX451149
P. areolata **Trel. aff.**	Pe922	*Samain et al.* 2009-189 (BR, GENT, USM); BG Gent, 2009 0772	KX451150
P. argyreia **(Miq.) Morr. aff.**	Pe175	*Symmank et al.* 2008-052 (BR, GENT, LPB)	KX451165
P. dolabriformis **Kunth aff.**	Pe915	*Samain 2013-078* (GENT); Ecuador; BG Gent, 2006 1014	KX451151
P. emarginulata **C.DC. aff.**	Pe913	*Mathieu et al*. 2009-161 (BR, GENT, USM); Peru; BG Gent; 2009 0606	KX451154
P. fenzlii **Regel aff.**	Pe893	*Samain 2013-081* (GENT); BG Gent, 1900 4097	KX451164
P. fissicola **Trel. aff. 1**	Pe768	*Samain et al.* 2010-197 (GENT, USM); BG Gent, 2011 0300	KX451155
P. fissicola **Trel. aff. 2**	Pe400	*Mathieu et al*. 2009-141 (BR, GENT, USM); BG Gent, 2009 0587	KX451166
P. galioides **Kunth**	Pe733	*Samain et al.* 2010-162 (GENT, USM)	KX451156
P. glabella **(Sw.) A.Dietr. aff.**	Pe899	*Samain* 2013-082 (GENT); Nicaragua, Jinotega; BG Gent, 2002 1190	KX451152
P. hispiduliformis **Trel. aff.**	Pe056	*Samain et al.* 2007-101 (BR, GENT, MEXU); Mexico, Oaxaca	KX451167
P. hylophila **C.DC. aff.**	Pe900	*Samain 2013-086* (GENT); BG Gent, 2001 2617	KX451153

P. ilaloensis **Sodiro aff.**	Pe891	*Frenzke* 007 (DR);<br>Peru, Cuzco;<br>BG Gent, 2003 1344	KX451157
P. inaequalifolia **Ruiz & Pav. aff.**	Pe917	*Samain* 2013-087 (GENT);<br>Paraguay, Alto Paraná;<br>BG Gent, 2004 1988	KX451158
P. leptostachya **Hook. & Arn.**	Pe934	*Frenzke* 006 (DR);<br>Pitcairn Islands;<br>BG Gent, 2002 2238	KX451171
P. macrothyrsa **Miq. aff.**	Pe897	*Mathieu et al*. 2009-144<br>(BR, GENT, USM);<br>Peru, Cajamarca;<br>BG Gent, 2009 0590	KX451159
P. obtusifolia **var.** ***emarginata***	Pe910	*Pino* 1101 (USM);<br>Peru, Piura;<br>BG Gent, 2007 0805	KX451160
P. praeruptorum **Trel. aff.**	Pe895	*Samain et al.* 2009-222<br>(BR, GENT, USM);<br>Peru, Huánuco;<br>BG Gent, 2009 0805	KX451161
P. sodiroi **C.DC. aff.**	Pe932	*Laegaard* 19853 (AAU);<br>Ecuador, Loja;<br>BG Gent, 2003 2008	KX451168
P. species 1	Pe819	*Samain et al.* 2010-204<br>(GENT, USM);<br>Peru, Amazonas;<br>BG Gent, 2011 0037	KX451169
P. species 2	Pe193	*Symmank et al.* 2008-077<br>(GENT);<br>BG Gent, 2008 0280	KX451170
P. species 3	Pe919	*Mathieu et al*. 450 (GENT);<br>Madagascar;<br>BG Gent, 2001 2551	KX451162
P. species 4	Pe901	*Samain* 2013-101 (GENT);<br>Colombia;<br>BG Gent, 2007 0777	KX451163

Table S2 Regions excluded from phylogenetic analyses because of uncertain sequence homology (hotspots).

hotspot	position
H1	4357 –4418
H2	3997–4014
H3	3856–3920
H4	3750–3770
H5	3662–3668
H6	3624–3644
H7	3424–3511
H8	3319–3360
H9	3279–3303
H10	3079
H11	2985–3043
H12	2544–2552
H13	1964–2014
H14	1930–1936
H15	1534–1547
H16	1190–1227
H17	1138–1152
H18	1006–1009
H19	729–738
H20	688–695
H21	1–54

Table S3 Matrix of sampled taxa and their corresponding character states: life form (0 – terrestrial, 1 – epiphytic), fruit stickiness (0 – absent, 1 – present), pseudopedicel (0 – absent, 1 – present), fruit apex (0 – pointed, 1 – beaked).

**species**	**origin of fruit samples**	**life form**	**fruit stickiness**	**pseudopedicel**	**fruit apex**
P. abyssinica **Miq.**	BG Gent, 1975 3227	0	1	1	1
P. aceramarcana **Trel.**	BG Gent, 2008 1231	1	1	1	0
P. alata **Ruiz & Pav.**	BG Gent, 2005 1342	?	1	1	1
P. alismifolia **Presl aff.**	BG Gent, 2011 0027	0	0	0	0
P. alpina **(Sw.) A.Dietr. aff.**	BG Gent, 2006 1279	1	1	0	1
P. andina **Pino**	Peru, Cajamarca	0	0	0	0
P. antioquiensis **Callejas**	BG Gent, 2010 1018	0	0	0	0
P. areolata **Trel. aff.**	BG Gent, 2009 0772	0	1	0	1
P. argyreia **(Miq.) Morr. aff.**	BG Gent, 2008 0383	0	0	0	0
P. arifolia **Miq.**	BG Gent, 2007 1717	0	0	0	0
P. bicolor **Sodiro**	BG Gent, 2003 1986	0	1	1	1
P. bracteata **A.W.Hill**	BG Gent, 2007 1270	0	0	0	0
P. clusiifolia **(Jacq.) Hook.**	BG Gent, 1987 2128	?	1	0	1
P. columella **Rauh & Hutchison**	BG Gent, 2007 0811	0	0	0	0

***P. cotyledon* Benth.**	BG Gent, 2002 2158	0	0	0	0
***P. cuspidilimba* C.DC.**	BG Gent, 2003 1616	1	1	1	0
***P. cymbifolia* Pino**	BG Gent, 2009 0487	0	0	0	0
***P. dahlstedtii* C.DC.**	BG Gent, 2003 1671	1	1	1	0
***P. dependens* Ruiz & Pav.**	BG Gent, 2009 0556	0	0	0	0
***P. deppeana* Schltdl. & Cham.**	BG Gent, 2002 2045	1	1	0	0
***P. dolabella* Rauh & Kimnach**	priv. collection R. Mayer	0	0	0	0
***P. dolabriformis* Kunth**	BG Gent, 2006 1303	0	0	0	0
***P. dolabriformis* Kunth aff.**	BG Gent, 2006 1014	0	0	0	0
***P. dolabriformis* var. *lombardii* Pino**	BG Gent, 2011 0020	0	0	0	0
P. elongata	BG Gent, 2004 1471	1	1	0	1
***P. emarginella* (Sw. ex. Wikstr.) C.DC.**	BG Gent, 2002 1923	1	0	0	?
***P. emarginulata* C.DC. aff.**	BG Gent, 2009 0606	0	1	1	1
***P. exclamationis* G. Mathieu**	México, Michoacán	0	0	0	0
***P. fagerlindii* Yunck.**	BG Gent, 1900 4158	1	1	0	1
***P. fenzlii* Regel**	BG Gent, 1972 1167	0	1	1	1
***P. fenzlii* Regel aff.**	BG Gent, 1900 4097	0	1	1	0

***P. fissicola* Trel. aff. 1**	BG Gent, 2011 0300	0	0	0	0
***P. fissicola* Trel. aff. 2**	BG Gent, 2009 0587	0	0	0	0
***P. fraseri* C.DC.**	BG Gent, 1972 1079	0	0	0	0
***P. galioides* Kunth**	BG Gent, 1978 1274	0	1	1	1
***P. glabella* (Sw.) A.Dietr.**	BG Gent, 2003 1637	1	1	1	1
***P. glabella* (Sw.) A.Dietr. aff.**	BG Gent, 2009 0742	1	1	1	1
***P. gracillima* Wats.**	BG Gent, 2004 1123	0	0	0	0
***P. graveolens* Rauh & Barthlott**	BG Gent, 2007 0825	0	0	0	0
***P. hendersonensis* Yunck.**	BG Gent, 2002 2244	0	1	1	0
***P. hirta* C.DC.**	BG Gent, 2001 1340	1	1	0	1
***P. hispidula* (Sw.) A.Dietr.**	Costa Rica, Cartago	0	0	0	0
***P. hispiduliformis* Trel. aff.**	Mexico, Oaxaca	0	0	0	0
***P. hoffmannii* C.DC.**	BG Gent, 1972 2107	1	1	0	0
***P. humilis* A.Dietr.**	BG Gent, 2004 1478	0	1	1	0
***P. hylophila* C.DC.**	BG Gent, 1900 4024	0	1	1	1
***P. hylophila* C.DC. aff.**	BG Gent, 2001 2617	1	1	1	1
***P. ilaloensis* Sodiro aff.**	BG Gent, 2003 1344	0	1	1	1

***P. inaequalifolia* Ruiz & Pav. aff.**	BG Gent, 2004 1988	0	1	1	0
***P. incana* (Haw.) Hook.**	BG Gent, 2003 1628	1	?	0	1
***P. kimnachii* Rauh**	BG Gent, 2002 1210	1	1	1	0
***P. lanceolatopeltata* C.DC.**	BG Gent, 1900 4181	0	1	0	0
***P. lancifolia* Hook.**	BG Gent, 2007 1279	0	0	1	1
***P. lehmannii* C.DC.**	BG Gent, 2004 2110	0	?	0	0
***P. leptostachya* Hook. & Arn.**	BG Gent, 2002 2238	?	1	1	1
***P. longespicata* C.DC.**	BG Gent, 2003 1608	1	1	0	0
***P. macrorhiza* Kunth**	Perú, La Libertad	0	0	0	0
***P. macrostachya* (Vahl) A.Dietr.**	BG Gent, 2003 1681	1	1	0	0
***P. macrothyrsa* Miq. aff.**	BG Gent, 2009 0590	0	1	0	0
***P. maculosa* (L.) Hook.**	BG Dresden, 016235-17	0	1	0	1
***P. magnoliifolia* (Jacq.) A.Dietr.**	BG Gent, 1900 4058	?	1	0	1
***P. marmorata* Hook. f.**	BG Bonn, 17527	0	0	0	0
***P. maypurensis* Kunth**	BG Gent, 2003 1685	0	0	0	0
***P. metallica* Lind. & Rod.**	BG Gent, 2007 1718	1	0	0	0
***P. molleri* C.DC.**	BG Gent, 1900 4025	1	1	1	0

***P. nivalis* Miq.**	Peru, Cajamarca	0	0	0	0
***P. obtusifolia* (L.) A.Dietr.**	BG Gent, 2006 0237	?	1	0	1
P. obtusifolia* var. *emarginata	BG Gent, 2007 0805	?	1	0	1
***P. pallida* (Forst. f.) A.Dietr.**	BG Gent, 2002 2220	0	1	?	0
***P. pedunculata* C.DC.**	BG Gent, 1992 1945	0	1	1	0
***P. pellucida* (L.) Kunth**	BG Dresden, 016908-24	0	0	0	0
***P. pereskiifolia* (Jacq.) Kunth**	BG Gent, 008 0309	1	1	1	0
***P. pernambucensis* Miq.**	BG Gent, 1972 1039	0	1	0	0
***P. pinoi* G.Mathieu**	Peru, Huánuco	0	0	0	0
***P. pitcairnensis* C.DC.**	BG Dresden, 016907-23	?	1	1	0
***P. polystachya* (Ait.) Hook.**	BG Gent, 1900 3906	?	1	1	1
***P. ppucuppucu* Trel.**	BG Gent, 1900 3970	0	1	0	0
***P. praeruptorum* Trel. aff.**	BG Gent, 2009 0805	0	1	1	1
***P. procumbens* C.DC.**	BG Gent, 2009 0815	0	0	0	0
***P. prostrata* Williams**	BG Gent, 2003 1663	1	0	1	1
***P. pseudovariegata* C.DC.**	BG Gent, 2009 0780	0	1	1	1
***P. puberulispica* C.DC.**	BG Gent, 1971 0086	1	1	1	1

P. ratticaudata **G.Mathieu**	BG Gent, 2001 2594	?	1	0	0
P. reptilis **C.DC.**	BG Gent, 1973 0161	1	0	0	0
P. resediflora **Lind. & André**	BG Gent, 2007 0804	0	0	0	0
P. reticulata **Balf. f.**	BG Gent, 2002 1954	0	1	1	0
P. rhexiifolia **C.DC.**	BG Gent, 2007 1278	0	1	1	0
P. rhombea **Ruiz & Pav.**	BG Gent, 1900 3973	1	1	1	0
P. rotundifolia **(L.) Kunth**	BG Gent, 1900 4744	1	1	1	0
P. rotundilimba **C.DC.**	BG Gent, 2001 2530	1	1	1	1
P. rubella **(Haw.) Hook.**	BG Gent, 2003 1609	0	1	1	?
P. serpens **(Sw.) Loudon**	Bolivia, Cochabamba	1	0	0	1
P. societatis **Moore**	BG Gent, 2002 2227	0	1	1	0
P. sodiroi **C.DC. aff.**	BG Gent, 2003 2008	0	0	0	0
P. species 1	BG Gent, 2011 0037	0	0	0	0
P. species 2	BG Gent, 2008 0280	0	1	0	1
P. species 3	BG Gent, 2001 2551	?	1	1	0
P. species 4	BG Gent, 2007 0777	1	1	1	1
P. tetragona **Ruiz & Pav.**	BG Gent, 2009 0760	0	1	1	0

P. tetraphylla **Hook. & Arn.**	BG Gent, 2008 0637	?	1	1	0
P. tomentella **Trel. & Yunck.**	BG Gent, 2007 1701	0	0	0	0
P. trichophylla **Baker**	BG Gent, 2001 2511	1	1	1	1
P. trifolia **(L.) A.Dietr.**	BG Gent, 1997 0424	1	1	1	1
P. tristachya **Kunth**	Colombia, Cundinamarca	0	?	?	0
P. truncigaudens **C.DC.**	BG Gent, 2002 1193	1	1	0	1
P. tuerckheimii	BG Gent, 2007 1292	0	1	0	0
P. tuisana	BG Berlin, 173-24-95-33	1	1	1	?
P. urocarpa	BG Gent, 2009 1717	1	1	0	1
P. urocarpoides	BG Gent, 2007 1251	1	1	0	1
P. velutina	BG Gent, 2004 2073	0	1	1	1
P. verschaffeltii	BG Gent, 1900 3913	0	0	0	0
P. vestita	BG Gent, 2003 1696	0	?	0	1
P. vinasiana	BG Berlin, 173-25-95-33	1	0	0	0
P. vulcanica	BG Gent, 2003 1659	0	1	1	1

Table S4 Model comparisons tests of character evolution to identify the best-fitting model to explain our data on life form and fruit traits. Based on Akaike weights (w_i), the full BiSSE split model (in bold) was the best model, estimating transition and diversification rates for foreground and background lineages separately. Subscript numbers refer to character states: life form terrestrial (0) or epiphytic (1), fruit stickiness absent (0) or present (1), pseudopedicel absent (0) or present (1), fruit apex pointed (0) or beaked (1).

	No.	Model	No. parameters	Ln *L*	AIC	ΔAIC	w_i
**life form 0-terrestrial, 1-epiphytic**	**1**	**full BiSSE split**	**12**	**-118.91**	**261.82**	**0.00**	**0.951**
	2	full BiSSE	6	-129.85	271.69	9.87	0.010
	3	$\lambda_0 = \lambda_1$	5	-130.72	271.44	9.62	0.010
	4	$\mu_0 = \mu_1$	5	-131.96	273.93	12.11	0.002
	5	$q_{01} = q_{10}$	5	-281.178	572.35	310.53	< 0.001
	6	$\lambda_0 = 0$	5	-392.09	794.17	532.35	< 0.001
	7	$\lambda_1 = 0$	5	-309.36	628.71	366.89	< 0.001
	8	$\mu_0 = 0$	5	-131,69	273.39	11.57	0.003
	9	$\mu_1 = 0$	5	-129.85	269.69	7.87	0.020
	10	$q_{01} = 0$	5	-135.01	280.01	18.19	< 0.001
	11	$q_{10} = 0$	5	-182.36	374.72	112.90	< 0.001
	12	$\lambda_0 = \lambda_1, \mu_0 = \mu_1$	4	-131.68	271.36	9.54	0.008
**stickiness 0-absent, 1-present**	**1**	**full BiSSE split**	**12**	**18.96**	**-13.92**	**0.00**	**0.969**
	2	full BiSSE	6	6.50	-0.99	12.92	0.002
	3	$\lambda_0 = \lambda_1$	5	7.06	-4.12	9.79	0.007
	4	$\mu_0 = \mu_1$	5	7.30	-4.60	9.31	0.010
	5	$q_{01} = q_{10}$	5	7.14	-4.29	9.63	0.008
	6	$\lambda_0 = 0$	5	-32.67	75.34	89.25	< 0.001
	7	$\lambda_1 = 0$	5	-44.10	98.20	112.12	< 0.001
	8	$\mu_0 = 0$	5	2.13	5.73	19.65	< 0.001
	9	$\mu_1 = 0$	5	-36.50	82.99	96.91	< 0.001
	10	$q_{01} = 0$	5	6.50	-2.99	10.92	< 0.001
	11	$q_{10} = 0$	5	-22.57	55.14	69.05	< 0.001
	12	$\lambda_0 = \lambda_1, \mu_0 = \mu_1$	4	2.46	3.08	17.00	< 0.001

**pseudopedicel 0-absent, 1-present**	1	**full BiSSE split**	**12**	**21.53**	**-19.05**	**0.00**	**0.953**
	2	full BiSSE	6	12.46	-12.92	10.74	0.047
	3	$\lambda_0 = \lambda_1$	5	1.41	7.17	26.23	< 0.001
	4	$\mu_0 = \mu_1$	5	2.14	5.71	24.76	< 0.001
	5	$q_{01} = q_{10}$	5	3.11	3.78	22.83	< 0.001
	6	$\lambda_0 = 0$	5	-30.32	70.64	89.69	< 0.001
	7	$\lambda_1 = 0$	5	-47.00	104.00	123.05	< 0.001
	8	$\mu_0 = 0$	5	-3.54	17.07	36.13	< 0.001
	9	$\mu_1 = 0$	5	-3.15	16.30	35.35	< 0.001
	10	$q_{01} = 0$	5	-5.42	20.84	39.90	< 0.001
	11	$q_{10} = 0$	5	-19.99	49.98	69.03	< 0.001
	12	$\lambda_0 = \lambda_1$, $\mu_0 = \mu_1$	4	-2.26	12.52	31.58	< 0.001
**fruit apex 0-pointed, 1-beaked**	**1**	**full BiSSE split**	**12**	**-2.18**	**28.36**	**0.00**	**0.999**
	2	full BiSSE	6	-15.25	42.50	14.15	0.001
	3	$\lambda_0 = \lambda_1$	5	-19.95	49.90	21.55	< 0.001
	4	$\mu_0 = \mu_1$	5	-19.56	49.12	20.77	< 0.001
	5	$q_{01} = q_{10}$	5	-24.98	59.95	31.60	< 0.001
	6	$\lambda_0 = 0$	5	-33.63	77.26	48.90	< 0.001
	7	$\lambda_1 = 0$	5	-43.71	97.42	69.06	< 0.001
	8	$\mu_0 = 0$	5	-28.34	66.69	38.33	< 0.001
	9	$\mu_1 = 0$	5	-22.79	55.58	27.23	< 0.001
	10	$q_{01} = 0$	5	-19.66	49.33	20.97	< 0.001
	11	$q_{10} = 0$	5	-39.58	89.16	60.80	< 0.001
	12	$\lambda_0 = \lambda_1$, $\mu_0 = \mu_1$	4	-25.42	58.84	30.48	< 0.001
